# Cervical Dystonia Is Associated With Aberrant Inhibitory Signaling Within the Thalamus

**DOI:** 10.3389/fneur.2020.575879

**Published:** 2021-02-09

**Authors:** Christopher L. Groth, Mark Brown, Justin M. Honce, Erika Shelton, Stefan H. Sillau, Brian D. Berman

**Affiliations:** ^1^Department of Neurology, University of Iowa Hospitals and Clinics, Iowa City, IA, United States; ^2^Department of Neurology, University of Colorado Anschutz Medical, Aurora, CO, United States; ^3^Department of Radiology, University of Colorado Anschutz Medical, Aurora, CO, United States; ^4^Neurology Section, Denver VA Medical Center, Aurora, CO, United States

**Keywords:** cervical dystonia, GABA, thalamus, magnetic resonance spectroscopy, inhibition, positron emission tomography

## Abstract

**Objective:** The objective of this study is to investigate whether alterations in the neurotransmission of gamma-aminobutyric acid (GABA) in the thalamus are present in patients with cervical dystonia compared to healthy controls.

**Methods:** GABA magnetic resonance spectroscopy was used to investigate concentration levels of GABA in the thalamus of cervical dystonia patients (*n* = 17) compared to healthy controls (*n* = 18). Additionally, a focused *post hoc* analysis of thalamic GABA_A_ receptor availability data in a similar cohort (*n* = 15 for both groups) using data from a previously collected ^11^C-flumazenil positron emission tomography study was performed. Group comparisons for all evaluations were performed using two-sided *t*-tests with adjustments for age and sex, and Bonferroni correction for multiple comparisons was applied. Spearman's coefficient was used to test correlations.

**Results:** We found significantly reduced GABA+/Cre levels in the thalamus of cervical dystonia patients compared to controls, and these levels positively correlated with disease duration. Although mean thalamic GABA_A_ receptor availability did not differ between patients and controls, GABA_A_ availability negatively correlated with both disease duration and dystonia severity.

**Conclusions:** These findings support that aberrant inhibitory signaling within the thalamus contributes to the pathophysiology of cervical dystonia. Additionally, these results suggest that an inadequate ability to compensate for the loss of GABA through upregulation of GABA_A_ receptors may underlie more severe symptoms.

## Introduction

Isolated cervical dystonia (CD) is an adult-onset focal dystonia characterized by involuntary and sustained muscle contractions leading to abnormal postures and/or tremor of the neck (1). Inhibitory signaling deficiencies related to alterations in gamma-aminobutyric acid (GABA) have been previously evaluated using magnetic resonance spectroscopy (MRS) (2–4) and positron emission tomography (PET) with the radioligand ^11^C-flumazenil (5–7) in patients with adult-onset focal hand dystonia and cervical dystonia, respectively. Taken together, prior GABA MRS and ^11^C-flumazenil PET studies in isolated focal dystonia support that dystonia is associated with alterations in inhibitory signaling involving cortical, basal ganglia, and cerebellar regions of the brain. To date, however, no GABA MRS studies in dystonia patients other than focal hand dystonia have been reported, and no studies have evaluated both GABA levels and GABA_A_ availability in a specific region of interest in an isolated focal dystonia cohort.

In this study, we investigated GABA concentration levels within the right thalamus using GABA MRS. We further performed a *post hoc* analysis of data previously collected using ^11^C-flumazenil PET to specifically investigate GABA_A_ receptor availability within the bilateral thalamus of CD patients and controls using automated segmentation methods to define a subject-specific thalamic volume for a region of interest analysis as opposed to the previously reported whole-brain voxel-wise analysis. The thalamus was chosen for this investigation because it has been unexplored via MRS in dystonia to date. It is a region where GABAergic neuronal projections from the basal ganglia and cerebellum converge (8), has a significant role in efferent GABAergic signaling (9), and plays a central involvement in sensorimotor processing (10–12). We hypothesized that GABA levels in the right thalamus and GABA_A_ receptor availability in the bilateral thalamus would be reduced in patients with CD compared to healthy controls. Additionally, as an exploratory analysis, we evaluated for any correlations between inhibitory signaling within the thalamus to clinical features in CD patients.

## Methods

### Participants

Seventeen CD patients and 18 healthy controls (HC) were recruited for the GABA MRS study. Participants who underwent the previously reported ^11^C-flumazenil PET study included 15 CD patients and 15 healthy controls (6). Six CD patients and six HC participated in both imaging studies. For the initially recruited subjects, there were no significant differences in age, sex, and MoCA scores between subjects and controls nor in the dystonia duration or severity between subjects in the two imaging studies. Additionally, after removing subjects for exceeding error thresholds for the two studies, the demographics of the analyzed subjects remained similar as shown in Table 1 apart from a now significant difference in Toronto Western Spasmodic Torticollis Rating Scale (TWSTRS)-motor between the two dystonia studies.

**Table 1:** Demographics of analyzed participants for thalamic MRS and PET imaging studies.

	**Gamma-aminobutyric acid (GABA) MRS**			^****11****^**C-Flumazenil PET**			**MRS vs. PET**
	**CD**	**HC**	***P*-value**	**CD**	**HC**	***P*-value**	**P-value**
	**(*N* = 15)**	**(*N* = 16)**		**(*N* = 11)**	**(*N* = 13)**		**(CD/HC)**
Age, years	62.7 ± 9.3	66.2 ± 5.6	0.22	62.5 ± 8.4	64.9 ± 8.8	0.51	0.96/0.64
Sex, F:M	11:4	12:4	0.92	9:2	9:4	0.50	0.63/0.74
MoCA	28.7 ± 1.6	28.2 ± 1.7	0.43	28.7 ± 1.5	27.8 ± 1.5	0.13	0.92/0.49
Duration, years	13.2 ± 7.7	—	—	21.3 ± 14.3	—	—	0.08/—
TWSTRS—total	23.3 ± 10.8	—	—	18.5 ± 6.4	—	—	0.20/—
TWSTRS—motor	14.8 ± 5.3	—	—	10.7 ± 3.5	—	—	0.04/ —
GABAergic meds, Y:N	5:10	—	—	3:8	—	—	0.75/—
Head tremor, Y:N	10:5	—	—	3:8	—	—	0.05/—
Botulinum toxin, Y:N	11:4	—	—	7:4	—	—	0.61/—

*CD, cervical dystonia; HC, healthy controls; MoCA, Montreal Cognitive Assessment; TWSTRS—total, Toronto Western Spasmodic Torticollis Rating Scale total score; TWSTRS—motor, Toronto Western Spasmodic Torticollis Rating Scale motor sub-score; PET, positron emission tomography; MRS, magnetic resonance spectroscopy; GABAergic meds (clonazepam, primidone, or zolpidem)*.

Inclusion criteria for both studies included age of at least 18 years old, normal neurologic examination except for CD symptoms in affected individuals, and no cognitive impairment based on a score of ≥26 on the Montreal Cognitive Assessment score (MOCA) (13). CD patients were excluded if dystonia symptoms were potentially due to secondary causes (i.e., drug induced, structural lesion, etc.). We were unable to recruit solely GABAergic medication or botulinum toxin naive patients given that botulinum toxin injections are the gold standard of treatment in cervical dystonia as well as the goal to recruit subjects with varying severity and duration of dystonia and not just newly diagnosed after recent onset.

Dystonia severity was assessed using the TWSTRS (14). Of the CD patients receiving botulinum toxin injections, all were scanned when the botulinum toxin was felt to have effectively worn off and no sooner than 10 weeks following their last set of injections to potentially mitigate any GABA changes related to the injections (15, 16). Healthy participants taking GABAergic medication were excluded. CD patients felt to be at low risk for withdrawal symptoms from refraining from taking low, or as-needed, doses of a GABAergic medication (clonazepam: *n* = 4 and primidone: *n* = 1) were scanned at a minimum of five half-lives after cessation of medication. This study was carried out in accordance with the recommendations of the Colorado Multiple Institutional Review Board with written informed consent from all subjects. All subjects gave written informed consent in accordance with the Declaration of Helsinki.

### MRS Acquisition

**Figure 1:**
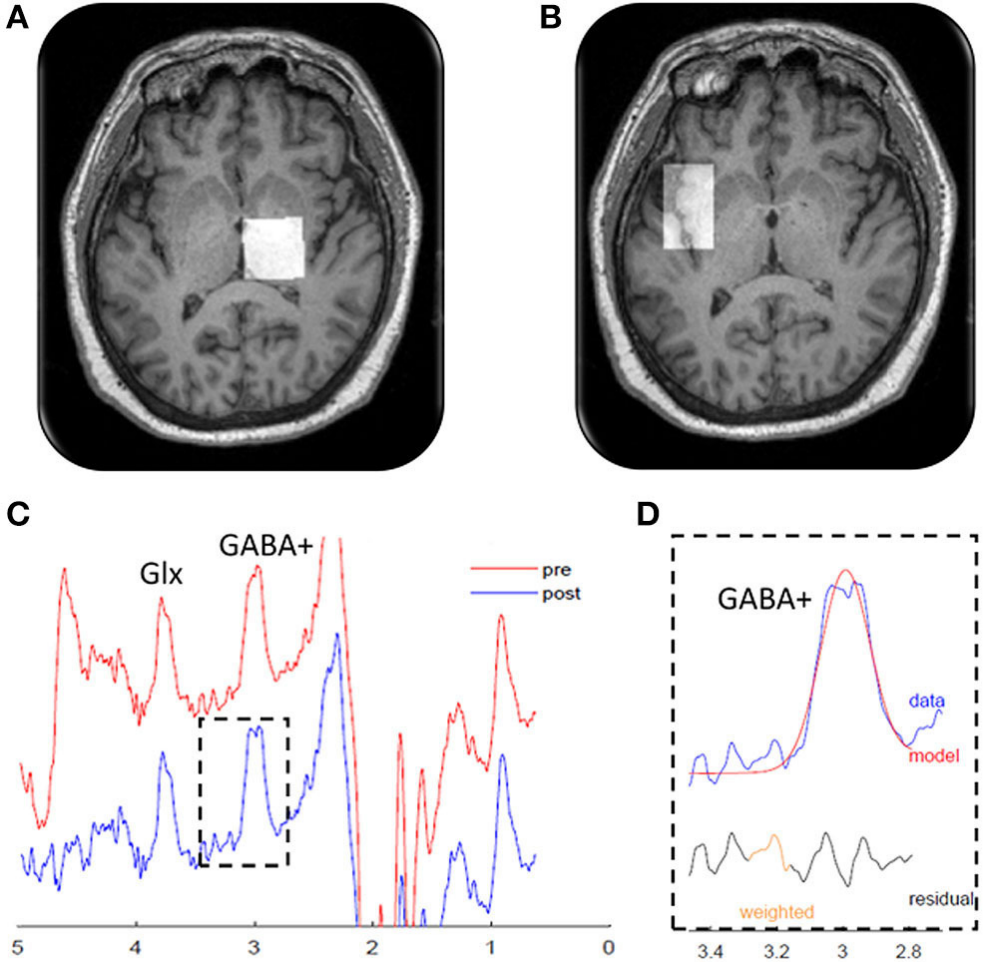
Magnetic resonance spectroscopy measurements. Localization of the magnetic resonance spectroscopy voxels (white squares) in the **(A)** right thalamus and **(B)** left insula. **(C)** Edited magnetic resonance spectroscopy spectrogram from a representative patient showing gamma-aminobutyric acid (GABA)+ and glutamine + glutamine (Glx) peaks. The red line shows the raw data, and the blue line shows the processed, frequency-aligned GABA+ data. The outlined portion **(D)** shows the fit output for the GABA+ signal. Residual refers to the difference between the observed data value and the model predicted value. Weighted refers to program modeling to downweight any choline subtraction artifact.

GABA MRS was performed using a Skyra 3T MRI system (Siemens Healthineers) using a 20-channel head/neck array. Pads, pillows, Velcro straps, and manipulation of body position were used to limit movement from tremor and enable the head to be positioned in the most neutral position possible. MRI scanning included a high-resolution T1-weighted anatomical scan (3D IR-prepped MPRAGE TR/TE/TI 2300/2.24/900 ms, flip angle 8°, FOV 256 × 256 mm, slice thickness 1 mm, 192 slices, matrix size 256^2^, phase direction R/L, GRAPPA = 2 and IPAT = 4) to facilitate the spectroscopy voxel placements. One MRS voxel (18.8 cm^3^) was placed over the right thalamus. The voxel was placed ~ 2 cm anterior to the internal auditory canal and placed to minimize inclusion of the lateral and fourth ventricles and to exclude the hippocampus (Figure 1A). A second MRS voxel (19.2 cm^3^) was placed over the left insula to serve as a control region and was centered on the largest gray matter slice, angled from the anterior to posterior insula (Figure 1B).

MRS acquisition of short echo-time water-suppressed PRESS spectra for both voxels was performed using the following parameters: TR/TE = 2,000/30 ms, 96 NAVs, receiver bandwidth 2,500 Hz, with eight phase cycling steps, time = 3:22. “J-edited” spectra for GABA determinations were acquired using a MEGA-PRESS sequence developed as a Work-in-Progress by Siemens Healthineers (17). The sequence features a center frequency update (“RFA”), which was applied every 10 averages to correct for center frequency drift due to possible warm magnet passive shim elements cooling during the acquisition. We have observed that the “RFA” frequency correction approach can occasionally overcorrect and then oscillate between two values if the cooling is intensive. In this way, the proper frequency for the editing pulses was maintained throughout the acquisitions. The editing pulses were applied at the editing frequency (1.9 ppm, “edit-on”) alternating with the pulses being applied on the other side of the water frequency (“6.6 ppm, edit-off”) to minimize baseline distortions between the two acquisitions. The editing pulses were Gaussians with a linewidth (FWHM) of 46 Hz. The parameter for the MEGA-PRESS acquisitions were TR/TE 2000/68 ms, bandwidth = 1,000 Hz, a total of 384 averages (192 edit-on and 192 edit-off), 4 prep (dummy) scans, time = 12:56. The data were exported in RDA formats for further analysis.

### MRS Analysis

Levels of the ratio of glutamate/creatine (Glu/Cre), and levels of glutamate + glutamine/creatine (Glx/Cre) were determined using PRESS data spectra. The data were processed using LCModel (version 6.2) and an appropriate basis set (18). Metabolite levels with Cramér-Rao bounds of <15% were considered. Concentrations for Glu/Cre and Glx/Cre and all other metabolites measured are reported in institutional units (IU). MEGA-PRESS spectra generated for the detection of GABA were processed using the Gannet 3.0 package of MATLAB scripts provided by Johns Hopkins University (19). Since we did not determine the contributions to the GABA signal from coedited macromolecules, the reported GABA values reflect GABA+ rather than pure GABA and are reported as GABA+/Cre ratios. Gannet 3.0 is also able to segment brain tissue (gray and white matter) from CSF, and all reported values were adjusted for possible CSF inclusion within the voxel.

All data were required to have a minimum fit error of <15% for inclusion in our data analysis. Additionally, all MRS data were reviewed and visually inspected by an expert in MRS (M.S.B.) and rejected if the MRS spectra (1) did not have discernable peaks coinciding with the neurotransmitters of interest, (2) was saturated with a signal indicative of considerable fat within the voxel of interest, suggesting voxel placement was suboptimal, or (3) if the spectra showed artifacts suggestive of motion (i.e., tremor), water suppression baseline distortion, excessive linewidths (poor shimming), or other factors such as the oscillatory behavior due to the frequency correction RFA mentioned above. Representative MRS spectra from MEGA-PRESS data after “j-editing” for isolation of GABA+/Cre are shown in Figures 1C,D.

### PET Acquisition

^11^C-flumazenil PET imaging data were acquired using previously reported methods (6). In brief, subjects were scanned on a Philips Gemini 64TF PET/CT imaging system (Philips Medical Systems, The Netherlands). An intravenous 20-mCi bolus of ^11^C-flumazenil was administered followed by a 60-min dynamic PET scan. PET images were acquired as 25 sequential three-dimensional frames of the entire brain, and the resultant data were reconstructed using a LOR-RAMLA algorithm for tissue attenuation correction and smoothed using a 5-mm full width at half maximum Gaussian kernel.

### PET Analysis

PET images for each participant were realigned to the mean volume. Any participants who after realignment were found to have mean translation >3 mm or rotation >3° were removed from further analysis. GABA_A_ binding availability was estimated from ^11^C-flumazenil PET data using previously reported methods (6). Briefly, we calculated voxel-by-voxel non-displaceable uptake binding potential (BP) with the QModeling toolbox (ver. 1.6.2) implemented in SPM8 using the basis function implementation of the two-step Simplified Reference Tissue Model 2 (20). The input kinetics for the reference tissue were derived from the pons, which has been used in prior ^11^C-flumazenil studies and found to be a reliable reference tissue that is highly correlated with the BP values estimated through arterial sampling (21). The pons was drawn for each participant separately using their anatomical MRI scan. In contrast to the previously reported whole-brain PET analysis, we extracted mean BP values from the processed PET images in individual subject space for all participants using a thalamic region of interest generated for each subject via an automated segmentation procedure described below.

### Volumetric Analysis

To assess for potential volumetric differences in the bilateral thalamus and create thalamic volumes for the current region of interest ^11^C-flumazenil PET analysis, we performed an automated segmentation analysis on the high-resolution anatomical MRI scan obtained on all participants. The MPRAGE images for each subject was first processed using the MINC (McConnell Brain Imaging Center, Montreal Neurological Institute) N3 tool to correct for signal intensity inhomogeneity across the images (22). Following this, the N3-corrected images were processed using the FIRST tool within the FMRIB Software Library (FSL) to extract volumes for the right and left thalamus of each participant (23). Visual inspection was performed, and any errors in segmentation were manually corrected within FSLView (by J.M.H, neuro-radiologist, who was blinded to the disease status of the subjects). All patient volumes were normalized to their skull size by utilizing the scaling factor output from the Structural Image Evaluation with Normalization of Atrophy Cross-sectional (SIENAX) tool within FSL (24).

### Statistical Analysis

Using SAS Version 9.4, group comparisons in patient demographics, GABA+/Cre levels, GABA_A_ receptor availability, and thalamic volumes were performed using two-sample two-tailed *t*-tests with regression adjusted for age and sex. Spearman's coefficient was used to test for partial correlations as Spearman's can detect any monotonic trend given uncertainty in linearity of data. Exploratory analysis with partial correlations were performed between right thalamic GABA+/Cre levels and GABA_A_ receptor binding in the bilateral thalamus to dystonia duration and severity. Furthermore, in subjects who had undergone both MRS and PET analysis, partial correlations were performed between GABA+/Cre levels in the right thalamus and GABA_A_ receptor binding. Results were adjusted for age and sex by using them as covariates in the regression and partial correlations. Significance was defined as *p* ≤ 0.05. Bonferroni correction for multiple comparisons was applied to the GABA spectroscopy and ^11^C-flumazenil PET data only for comparison of measured GABA values vs. healthy controls. The rest of the analyses performed were exploratory in nature and not primarily hypothesis driven. Thus, corrections were not applied to the exploratory data.

## Results

### GABA Levels in CD

GABA+/Cre levels were initially significantly lower in CD patients compared to HC in the right thalamus (CD: 0.116 ± 0.005, HC: 0.137 ± 0.007; *p* = 0.02). However, when performing Bonferroni correction for multiple comparisons, the finding of reduced GABA/Cre+ levels falls slightly outside of statistical significance (*p* > 0.0125). GABA+/Cre levels in the left insula were not significantly different (0.084 ± 0.006 vs. 0.091 ± 0.007, *p* = 0.41). Explorary analysis of levels of additional neurometabolites from our PRESS analysis are shown in Table 2. Of the additional neurometabolites measured, statistically significant decreases in myoinositol/Cre (*p* = 0.05) and glycerophosphocholine + phosphocholine/Cre (*p* = 0.03) were seen in the thalamus of CD patients compared to HC. There were no significant differences found in the creatine levels or any other neurometabolites. There were no statistical differences between groups in the amount of brain parenchyma included in the thalamic MRS voxel (CD: 92.3%, HC: 91%, *p* = 0.23) or the insula MRS voxel (CD: 77.4%, HC: 77.9%, *p* = 0.80).

**Table 2:** Additional neurometabolite level comparisons between patients with cervical dystonia and healthy controls.

	**Thalamus**			**Insula**		
	**CD (*n* = 17)**	**HC (*n* = 18)**	***P*-value**	**CD (*n* = 17)**	**HC (*n* = 18)**	***P*-value**
Cre	5.89 ± 0.16	5.75 ± 0.16	0.51	7.02 ± 0.25	6.74 ± 0.23	0.38
Glu/Cre	0.90 ± 0.04	0.84 ± 0.02	0.19	1.14 ± 0.03	1.11 ± 0.02	0.37
Glx/Cre	0.97 ± 0.05	0.90 ± 0.05	0.25	1.18 ± 0.04	1.15 ± 0.03	0.45
mI/Cre	0.71 ± 0.02	0.78 ± 0.04	0.05^*^	0.74 ± 0.03	0.81 ± 0.03	0.06
GPC+PCh/Cre	0.29 ± 0.01	0.31 ± 0.01	0.03^*^	0.29 ± 0.01	0.30 ± 0.01	0.20
NAA+NAAG/Cre	1.51 ± 0.02	1.53 ± 0.04	0.66	1.29 ± 0.04	1.31 ± 0.03	0.70

*Mean and standard deviations for neurometabolite concentrations shown. Values compared using two-tailed t-tests with adjustment for age and sex*.

* *Significant difference defined as p ≤ 0.05. Cre, creatine; GABA, gamma-aminobutyric acid; Glu, glutamate; Glx, glutatemate + glutamine; GPC, glycerophosphocholine; Lac, lactate; mI, myoinositol; NAA, N-acetylaspartate; NAAG, N-acetylaspartylglutamine; PCh, phosphocholine*.

**Figure 2:**
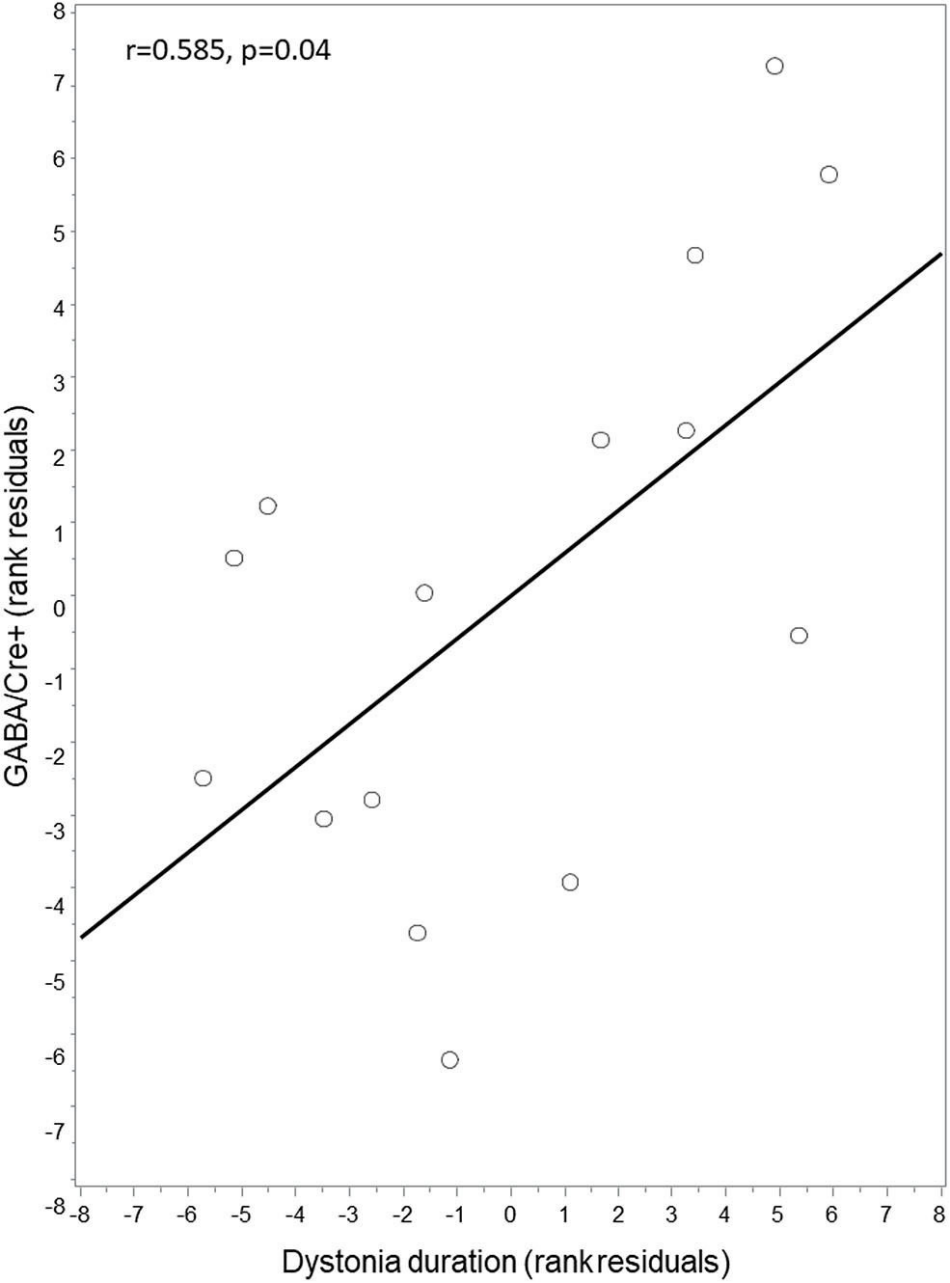
Correlation between thalamic GABA+/Cre and dystonia duration. Thalamic GABA+/Cre concentration levels were positively correlated with disease duration. Residuals are shown as data were adjusted for age and sex.

Exploratory analysis with partial Spearman correlation testing between right thalamic GABA+/Cre levels and clinical features of disease duration and motor severity (TWSTRS motor subscore) revealed a positive correlation between GABA+/Cre and duration of dystonia (*r* = 0.585, *p* = 0.04) (Figure 2). GABA+/Cre levels did not correlate with motor severity as measured by TWSTRS motor subscores (*r* = −0.348, *p* = 0.24). No significant correlations were found between insular GABA+/Cre levels and the clinical features of disease duration (*r* = −0.10, *p* = 0.75) or severity (*r* = −0.21, *p* = 0.49).

Furthermore, in consideration of possible contribution of tremor or medical therapy with GABAergic medications to GABA levels, a dystonia subject exploratory subgroup comparison of the thalamus showed no difference in GABA+/Cre levels between subjects with (*N* = 10) or without tremor (*N* = 5) (0.1211 ± 0.007 vs. 0.1212 ± 0.007, *p* = 0.99) or in those who did (*N* = 5) or did not (*N* = 10) take GABAergic medications (0.110 ± 0.01 vs. 0.118 ± 0.006, *p* = 0.50).

### Thalamic GABA_A_ Receptor Binding in CD

**Figure 3:**
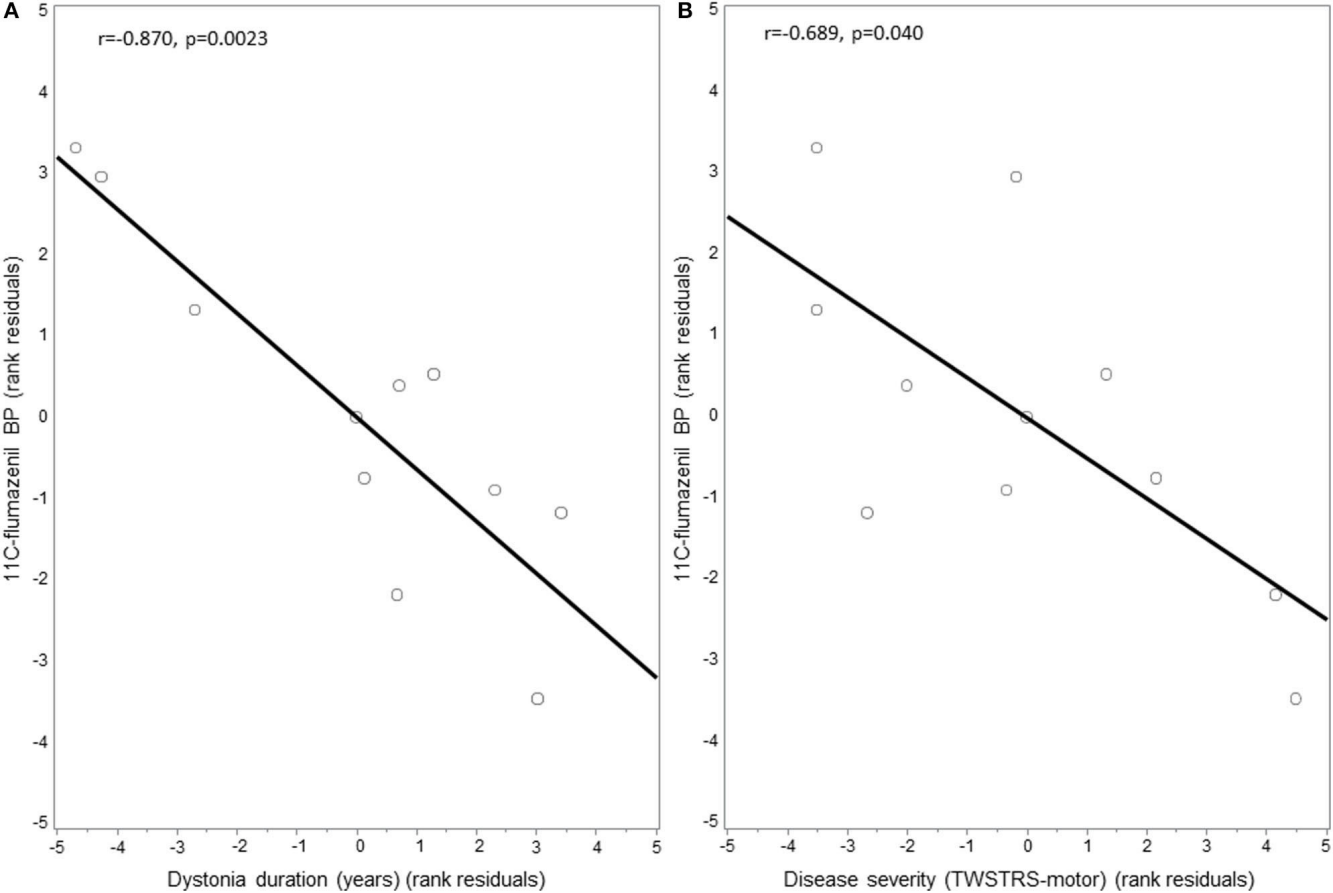
Correlation between thalamic 11C-flumazenil binding potential and dystonia motor features. GABAA receptor availability as measured by the binding potential (BP) of 11C-flumazenil in the thalamus of cervical dystonia patients was negatively correlated with: **(A)** disease duration and **(B)** dystonia severity as measured using the motor score of the Toronto Western Spasmodic Torticollis Rating Scale (TWSTRS). Residuals are shown as data were adjusted for age and sex.

GABA_A_ availability as measured by mean ^11^C-flumazenil PET BP did not significantly differ in the right thalamus in CD patients compared to HC (CD = 1.917 ± 0.217, HC = 1.799 ± 0.209; *p* = 0.435). Mean BP within the left thalamus similarly did not significantly differ between CD patients and HC (CD = 1.710 ± 0.156, HC = 1.635 ± 0.174; *p* = 0.524). GABA_A_ availability in the right thalamus, however, was negatively correlated with both disease duration (*r* = −0.870, *p* = 0.0023) (Figure 3A) and motor severity (*r* = −0.689, *p* = 0.040) (Figure 3B), while the left thalamus did not correlate with either disease duration or severity. Furthermore, in dystonia subject exploratory subgroup comparison of the right thalamus, there was no difference in PET BP levels between subjects with (*N* = 3) or without (*N* = 8) tremor (1.766 ± 0.200 vs. 1.824 ± 0.093, *p* = 0.78) or in those who did (*N* = 3) or did not (*N* = 8) take GABAergic medications (1.853 ± 0.063 vs. 1.867 ± 0.096, *p* = 0.88). An additional exploratory analysis was done to assess for any possible contribution of age to GABA_A_ receptor availability in the right thalamus. In evaluation of our healthy controls (*n* = 13), we found no significant correlation (*r* = 0.105, *p* = 0.733).

When doing an exploratory analysis of the six CD patients and the six HC who underwent imaging with both GABA MRS and ^11^C-flumazenil PET, no significant correlations were seen between GABA+/Cre and GABA_A_ availability in the right thalamus in either group (CD: *r* = −0.806, *p* = 0.19; HC: *r* = 0.205, *p* = 0.79).

### Thalamic Volumes

The volumes of the right thalamus estimated through our automated segmentation methods were compared between CD patients and HC across the two imaging study cohorts. No differences were found between the two groups in the GABA MRS cohort (CD: 9,982 ± 1,119 mm^3^, HC: 9,864.5 ± 657 mm^3^; *p* = 1.00) or the ^11^C-flumazenil PET cohort (CD: 10,125 ± 1,142 mm^3^, HC: 9,819 ± 1,017 mm^3^; *p* = 0.98). The volumes for the left thalamus also did not differ between the groups in the GABA MRS cohort (CD: 10,069 ± 967 mm^3^, HC: 10,185 ± 707 mm^3^; *p* = 0.19) or the ^11^C-flumazenil PET cohort (CD: 10,061 ± 942 mm^3^, HC: 10,039 ± 1,044 mm^3^; *p* = 0.30).

## Discussion

We report, for the first time, on reduced right thalamic GABA levels in patients with isolated adult-onset CD, though the significance did not hold up with Bonferroni correction. These findings, though not statistically significant, do suggest new evidence that inhibitory signaling within the thalamus is impaired in dystonia. Additionally, correlation between GABA levels in the right thalamus and disease duration suggests that GABAergic signaling may dynamically change over time in patients with CD. Although GABA_A_ receptor availability did not appear to differ between our CD cohort and HC, GABA_A_ availability correlated with both disease duration and severity in CD patients. Taken together, these data suggest a loss of GABA may be an early pathophysiological feature in CD and that insufficient postsynaptic compensatory response leads to the manifestation of more severe motor symptoms.

The thalamus serves as a critical structure for appropriate inhibitory signaling with integration of motor basal ganglia, cerebellar, and cortical circuits (8, 25). A large number of functional abnormalities of the thalamus have been reported in isolated focal dystonia, including CD (26–30). GABA is the main neurotransmitter used within the thalamic reticular nucleus and interneurons (31), which provide inhibitory innervation to the relay cells of the thalamus (32), and blocking GABA within the thalamus can lead to dystonic posturing in experimental models (33). GABAergic afferents from the internal segment of the globus pallidus also project inhibitory signals to the excitatory neurons within the ventrolateral nucleus of the thalamus and, as a result, lead to a reduction in activity in the motor cortex (34). Furthermore, the thalamus is a critical structure for sensory processing, which is known to be abnormal in CD (35–37) and can enhance and may even store sensory information (38). Thus, our finding of reduced GABA levels in the thalamus of CD patients is consistent with the hypothesis that the pathogenesis of CD stems from a loss of inhibitory control over the sensorimotor network and adds support to the concept of dystonia as a network disorder (39).

While we did not find a difference in bilateral thalamic GABA_A_ receptor availability between CD patients and healthy participants, our findings did reveal that lower right thalamic GABA_A_ availability was associated with increased motor severity. We also found a negative relationship between GABA_A_ receptor availability in the right thalamus and disease duration. Identifying possible inverse changes in GABA levels and GABA_A_ receptor availability over time in CD raises an intriguing hypothesis that there may be an adaptive process occurring in CD such that as reduced GABA levels tend to increase over time toward normal levels, and this increase in GABA concentration is associated with a concomitant decrease in GABA_A_ receptor availability. To best test this hypothesis would require longitudinal acquisition of GABA levels and GABA_A_ receptor availability in CD patients.

We further found no differences between bilateral thalamic volumes in CD patients and controls nor in amount of brain volume within the MRS voxels. Various advanced imaging studies with different protocols and different subgroups of dystonia have inconsistently shown changes in thalamic volume among other regions including the globus pallidus, putamen, and cerebellum (39, 40). Waugh et al. identified a decrease in thalamic volume in patients with CD, but did not find any alterations in the globus pallidus or putamen as have been previously identified as well (40). Thus, discussion remains as to the best method for potential identification of structural changes. FIRST has been shown to be comparable to other programs (i.e., FreeSurfer) in the measurement of the thalamus (41, 42), but continued work to optimize methods of structural assessment are still needed given the complexity in appropriately measuring the structure.

A potential explanation for the suggestive findings of reduced right thalamic GABA levels in CD that increase over time and the relationships we identified between GABA_A_ and the clinical features in CD patients could be that the pathogenesis of CD follows a “two-hit” hypothesis (43). In this case, the primary abnormality in adult-onset isolated CD before dystonia onset would be one of decreased GABA levels in the sensorimotor network that is accompanied by an increase in GABA_A_ receptors to maintain homeostasis in inhibitory signaling. The manifestation of dystonia then may arise if there is an insufficient increase in GABA_A_ receptors, or if a biological or other process occurs that leads to reduced GABA_A_ receptor availability.

Ultimately, further study of thalamic inhibitory changes in dystonia in context with its connections to the basal ganglia or cerebellar motor circuits is needed to better assess the larger network changes in inhibitory signaling that underlies the pathogenesis of CD. This is especially important given that this is the first study to evaluate the thalamus, and past studies have provided inconsistent results on GABA changes in the dystonia motor network given their marked variability in magnet strength, limited sample sizes, and variability in primary endpoints (2–4). As such, a consistent result over time would provide significant assurances in true inhibitory variances that have not been proven to date. Additionally, investigation into inhibitory signaling changes in those with CD, as well as those individuals at risk of CD—non-manifesting dystonia gene mutation carriers, first degree relatives of those with CD, and relatives of those with CD who have evidence of subclinical markers of gene carriage (44)—would further help delineate the inhibitory signaling abnormalities within the sensorimotor network and their role in the pathogenesis of CD.

This study has limitations. First, GABA MRS provides a quantitative assessment of GABA+/Cre concentration levels, but the technique is not able to distinguish between intracellular and extracellular GABA or discriminate between GABA in different cellular compartments. Past work suggests, however, that GABA MRS measures are likely reflective of neuromodulatory and neurotransmitter GABA stores (45). Additionally, GABA MRS is not able to directly measure GABA levels. Rather, GABA+ is measured in reference to another molecule, such as creatine. However, in our study, there was no statistical difference in creatine levels between CD patients and HC, which supports that our difference in GABA+/Cre level is driven by alterations in GABA levels and not by a difference in the reference molecule. Another limitation is that GABA MRS requires use of a large acquisition voxel such that our GABA+/Cre measurements may include brain tissue that lies outside the targeted brain structure. We limited the impact of this issue by aligning and orienting each voxel in three dimensions using each participant's high-resolution anatomical scan. A further limitation involves the recruitment of subjects receiving treatment for their dystonia including GABAergic medications and botulinum toxin injections. For patients with dystonia, botulinum toxin treatments have been shown to alter subcortical structures (46) as well as sensorimotor network activation (47) in response to treatment. Thus, we attempted to reduce potential for the treatments to affect GABA measurements as described in the Methods section, but exclusion of subjects receiving any form of treatment would limit our recruitment to subjects with only mild or short duration of symptoms and would markedly limit the applicability of the findings. Our exploratory subgroup analyses comparing dystonia subjects recently exposed to GABAergic medications to those without such exposure did not show any differences, suggesting that these medications did not likely alter our findings. However, this analysis is limited by small sample sizes. Future studies assessing the potential effect of these treatments on GABA levels and receptor availability in dystonia are needed to better assess if such an effect exists. Finally, the GABA MRS data and ^11^C-flumazenil PET data were collected on different cohorts at different timepoints and with a limited number of subjects undergoing both imaging studies limiting the reliability of our comparisons between the groups. Evaluating bilateral GABA levels and GABA_A_ availability at the same time in larger cohorts of patients and controls could enable a more accurate, comprehensive investigation into GABAergic signaling alterations in CD.

## Conclusions

To the best of our knowledge, this is the first multimodal study of the GABAergic signaling within the thalamus of patients with isolated, adult-onset CD. We found suggestive evidence that CD is associated with reduced right thalamic GABA levels that change over the duration of the disease and that increased GABA_A_ receptor availability in the right thalamus of these patients is linked to milder dystonia severity. Given that the decreased GABA level identified did not remain statistically significant when correcting for multiple comparisons and that our other analyses are only exploratory, further work is needed to determine the validity of these findings with larger cohorts and whether these changes in thalamic GABA are specific to CD and whether their alterations may affect selection of various treatment options (48). Overall, our findings further support the conceptual model of dystonia as a network disorder and that aberrant inhibitory signaling within the sensorimotor network contributes to the pathophysiology of dystonia.

## Data Availability Statement

The raw data supporting the conclusions of this article will be made available by the authors, without undue reservation.

## Ethics Statement

The studies involving human participants were reviewed and approved by Colorado Multiple Institutional Review Board University of Colorado, Anschutz Medical Campus. The patients/participants provided their written informed consent to participate in this study.

## Author Contributions

CG was involved in the conceptualization, methodology, validation, formal analysis, investigation, data curation, writing the original draft, reviewing, and editing, visualization, and funding acquisition. MB was involved in the conceptualization, methodology, validation, and writing the original draft. JH was involved in the conceptualization, methodology, validation, formal analysis, and writing the original draft. ES was involved in the validation, writing the original draft, and project administration. SS was involved in formal analysis and writing of the original draft. BB was involved in the conceptualization, methodology, validation, formal analysis, investigation, resources, data curation, writing the original draft, reviewing and editing, visualization, supervision, and funding acquisition. All authors contributed to the article and approved the submitted version.

## Conflict of Interest

CG has received research grant support from the Dystonia Medical Research Foundation. BB has received research grant support from the Dana Foundation, NIH (NIH/NCATS Colorado CTSI Grant Number KL2 TR001080), Dystonia Coalition (receives the majority of its support through NIH grant NS065701 from the Office of Rare Diseases Research in the National Center for Advancing Translational Science and National Institute of Neurological Disorders and Stroke), and from Mary Rossick Kern and Jerome H. Kern. The remaining authors declare that the research was conducted in the absence of any commercial or financial relationships that could be construed as a potential conflict of interest.
